# Supplementary material for: The Characterization of a Novel Virus Discovered in the Yeast Pichia membranifaciens
Source: Viruses. 2022 Mar 13;14(3):594. doi: 10.3390/v14030594 (PMC8951182; doi:10.3390/v14030594)
Supplement: Supplementary file 1 [file viruses-14-00594-s001.zip › Table S1.pdf]

| **Virus/yeast** | **Abbreviation** | **Accession Number** |
|---|---|---|
| Saccharomyces cerevisiae virus L-BC | ScV-L-BC | NC_001641.1 |
| *Scheffersomyces stipitis* CBS6054 | Ss CBS6054 | NC_009045.1 |
| Tuber aestivum virus 1 | TAV-1 | NC_038698.1 |
| Torulaspora delbrueckii virus LA | TdV-L-A | MW174763.1 |
| Saccharomyces cerevisiae virus L-A | ScV-L-A | NC_003745.1 |
| Saccharomyces cerevisiae virus L-A-lus | ScV-L-Alus | JN819511.1 |
| Saccharomyces paradoxus virus L-A-45 | SpV-L-A45 | KY489963.1 |
| Saccharomyces kudriavzevii virus L-A1 | SkV-L-A1 | NC_032106.1 |
| Saccharomyces paradoxus virus L-A-62 | SpV-L-A62 | KY489968.1 |
| Saccharomyces paradoxus virus L-A-74 | SpV-L-A74 | KY489964.1 |
| Saccharomyces uvarum virus L-A-10560 | SuV-L-A10560 | KY489969.1 |
| Saccharomyces cerevisiae virus L-A-28 | ScV-L-A28 | KU845301.2 |
| Saccharomyces paradoxus virus L-A-21 | SpV-L-A21 | KY489962.1 |
| Red clover powdery mildew-associated totivirus 1 | RPaTV1 | NC_028480.1 |
| Red clover powdery mildew-associated totivirus 4 | RPaTV4 | LC075489.1 |
| Red clover powdery mildew-associated totivirus 6 | RPaTV6 | NC_028486.1 |
| Red clover powdery mildew-associated totivirus 7 | RPaTV7 | NC_028488.1 |
| Maize associated totivirus | MATV | MN428832.1 |
| Puccinia striiformis totivirus 5 | PsTV-5 | KY207365.1 |
| Maize-associated totivirus 3 | MATV3 | NC_036589.1 |
| Black raspberry virus F | BRVF | NC_009890.1 |
| Peach-associated virus 2 | PAV2 | MN905505.1 |
| Taro-associated totivirus L | TATL | MN119621.1 |
| Panax notoginseng virus B | PnV-A | NC_040551.1 |
| Ambrosiozyma totivirus A | AkV-A | MK231133.1 |
| Scheffersomyces segobiensis virus L | SsV-L | NC_038697.1 |
| Malassezia restricta virus | MrV40L | MN603497.1 |
| Erysiphe necator associated totivirus 5 | EnaTV5 | MN617026.1 |
| Xanthophyllomyces dendrorhous virus L1b | XdV-L1b | NC_038699.1 |
| Puccinia striiformis totivirus 1 | PsTV1 | KY207361.1 |
| Erysiphales associated totivirus 7 | EaTV7 | MN628278.1 |
