# Supplementary material for: The Characterization of a Novel Virus Discovered in the Yeast Pichia membranifaciens
Source: Viruses. 2022 Mar 13;14(3):594. doi: 10.3390/v14030594 (PMC8951182; doi:10.3390/v14030594)
Supplement: Supplementary file 1 [file viruses-14-00594-s001.zip › Table S2.pdf]

Model	Molprobity score	clashscore	Ramachandran favored	Ramachandran outliers	Rotamer outliers
PmV Gag	1.02	0	95%	3	9
PmV Pol	1.25	0.15	92.18%	7	13
ScV-L-A Pol	1.37	0.15	92.06%	5	19
TdV-LABarr1 Pol	1.38	0.82	90.20%	8	9
TAV1 Pol	1.24	0.22	92.89	6	13
