# Supplementary figures and images for: The Characterization of a Novel Virus Discovered in the Yeast Pichia membranifaciens
Source: Viruses. 2022 Mar 13;14(3):594. doi: 10.3390/v14030594 (PMC8951182; doi:10.3390/v14030594)

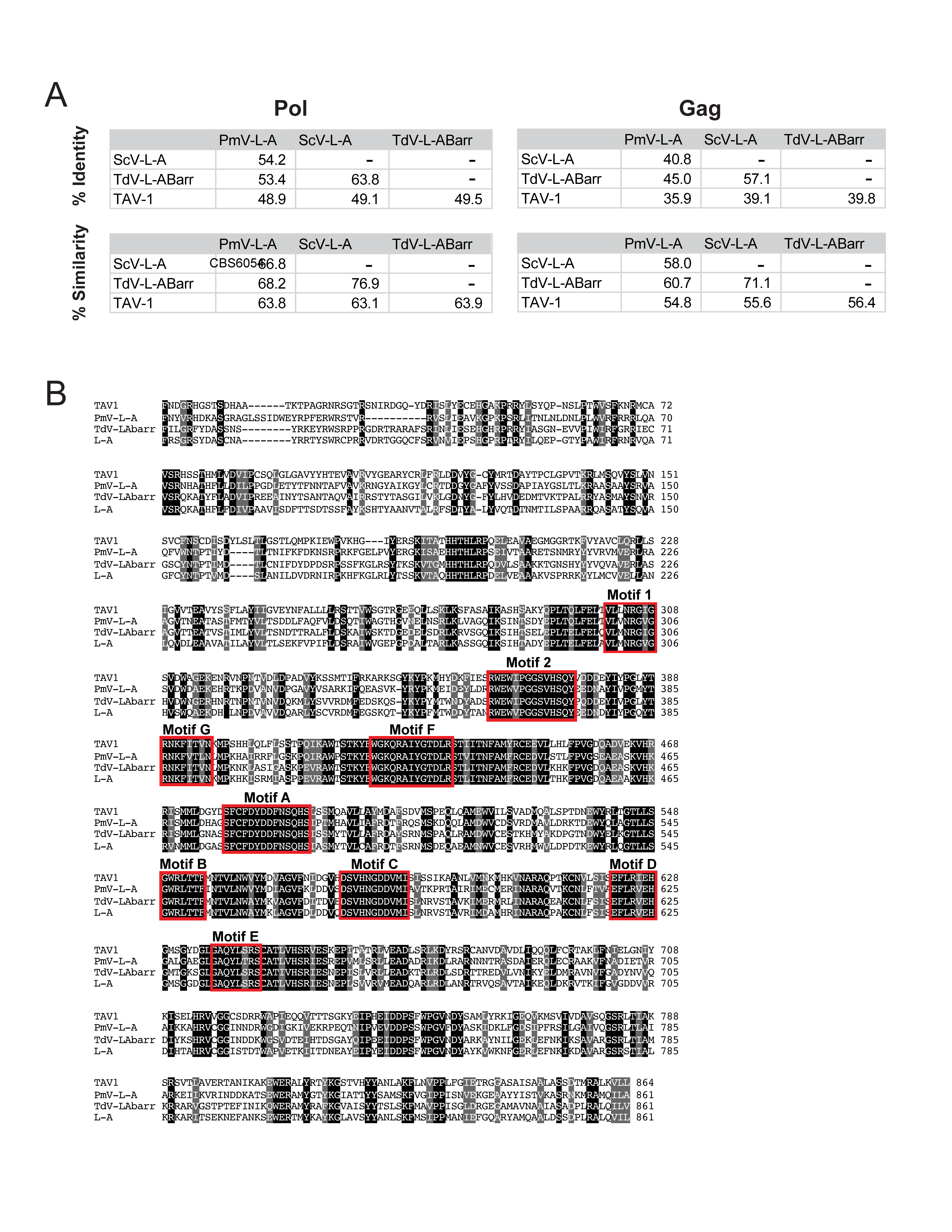

Supplement: Supplementary file 1 [file viruses-14-00594-s001.zip › Figure s3.tif]

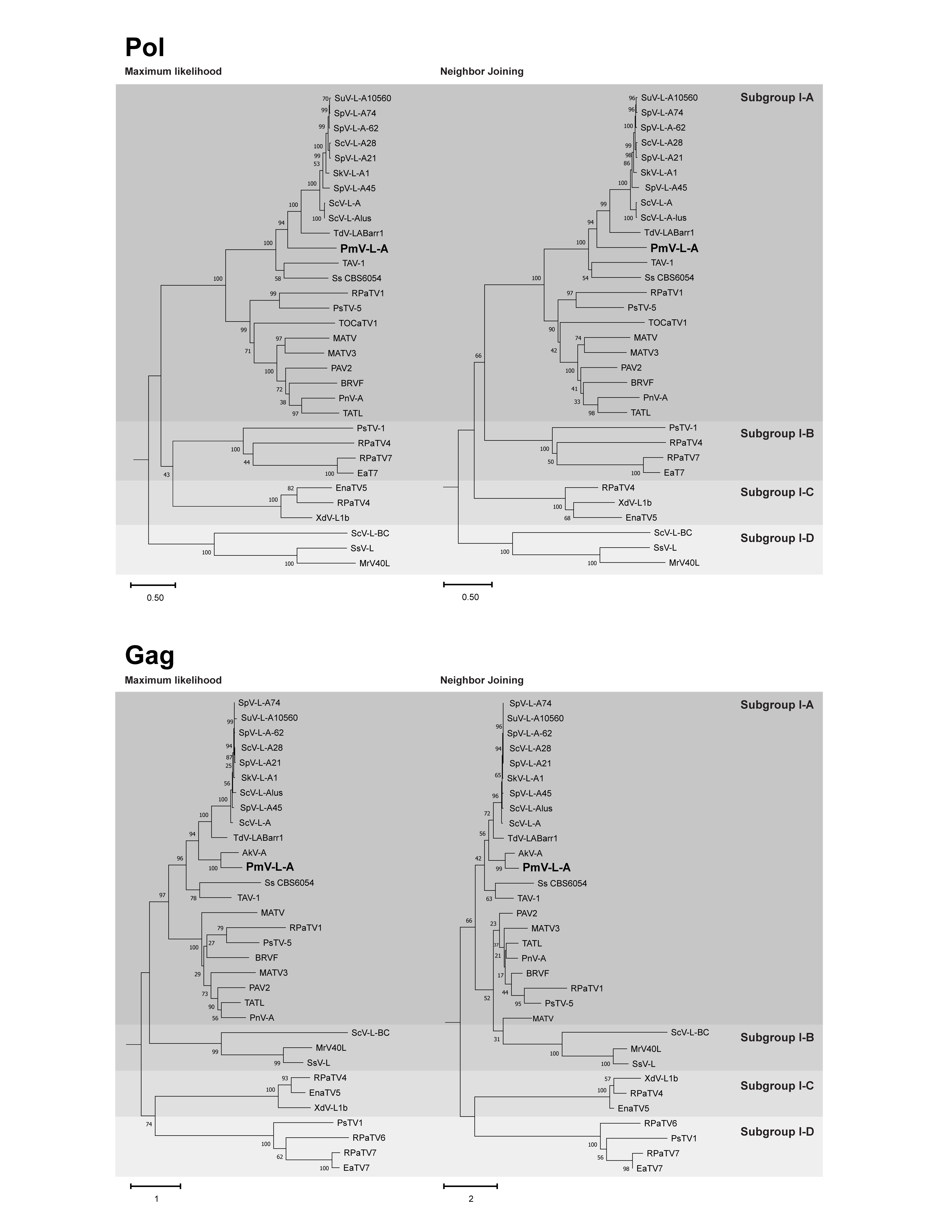

Supplement: Supplementary file 1 [file viruses-14-00594-s001.zip › Figure_s1.tif]

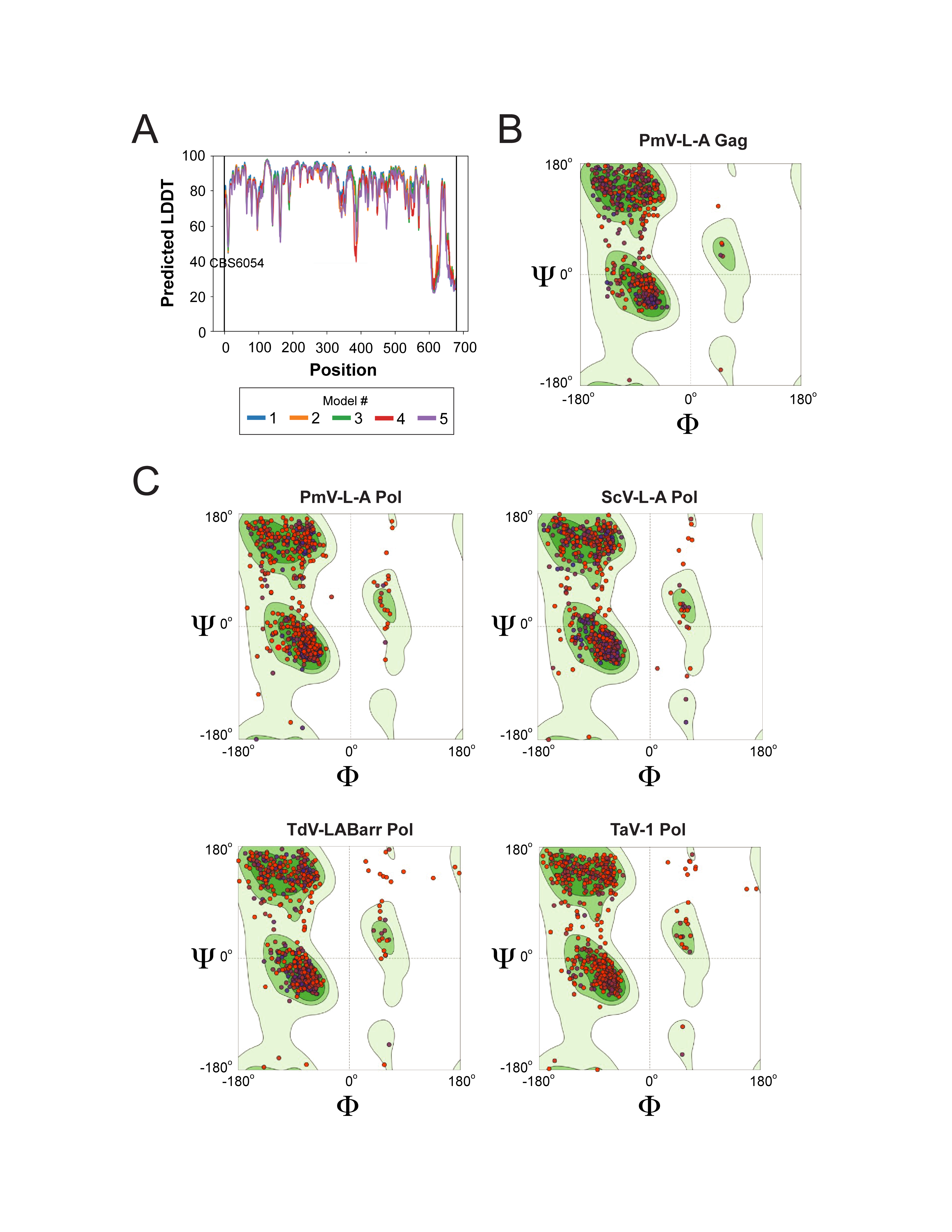

Supplement: Supplementary file 1 [file viruses-14-00594-s001.zip › Figure_s2.tif]
